# Comparison Between Respiratory‐Triggered and Free‐Breathing Signal Acquisition Methods for Single‐Voxel Phosphorus Magnetic Resonance Spectroscopy of the Liver on a Clinical System

**DOI:** 10.1002/jmri.70352

**Published:** 2026-05-02

**Authors:** Kumi Ozaki, Jihun Kwon, Marc Van Cauteren, Yasutomo Katsumata, Yukichi Tanahashi, Satoshi Goshima

**Affiliations:** ^1^ Department of Radiology Hamamatsu University School of Medicine Hamamatsu Japan; ^2^ Philips Japan Tokyo Japan

**Keywords:** adenosine triphosphate, inorganic phosphate, liver, phosphodiester, phosphomonoester, phosphorus magnetic resonance spectroscopy

## Abstract

**Background:**

The optimal protocol for clinical liver ^31^P‐magnetic resonance spectroscopy (MRS) remains unclear. Single‐voxel ^31^P‐MRS using image‐selected in vivo spectroscopy (ISIS) employs respiratory‐triggering (RT) or free‐breathing (FB) acquisition. RT provides robust data but prolongs scan duration; FB allows faster acquisition but may suffer from low signal‐to‐noise ratio (SNR). Yet, direct comparison using a regulatory‐approved coil in a clinical setting has not been reported.

**Purpose:**

To compare ^31^P‐MRS data stability and robustness between RT and FB.

**Study Type:**

Prospective.

**Population:**

24 volunteers (19 male/5 female).

**Field Strength/Sequence:**

3 T MRI; single‐voxel ^31^P‐MRS using ISIS (free induction decay‐based) with approved ^31^P coil.

**Methods:**

^31^P‐MRS was performed using RT and FB techniques (128 and 192 signal averages, respectively; expected scan duration ~13 min each). Spectra were analyzed using jMRUI. SNR and peak areas for PME, Pi, PDE, α‐ATP, and NADPH, normalized using γ‐ATP, were compared. PME/PDE and NADPH/(PME + PDE) ratios were also compared.

**Statistical Test:**

Paired *t*‐tests and Bland–Altman analysis were used. A *p* value < 0.05 was considered significant.

**Results:**

Scan duration was significantly longer for RT (15 min 44 s) than FB (12 min 56 s). No significant differences were observed for SNR (*p* = 0.570), NADPH/(PME + PDE) (*p* = 0.931), PME/γ‐ATP (*p* = 0.556), Pi/γ‐ATP (*p* = 0.931), α‐ATP/γ‐ATP (*p* = 0.332), or NADPH/γ‐ATP (*p* = 0.394). Significant differences were noted for PDE/γ‐ATP (RT 1.68 vs. FB 1.35, *p* = 0.003) and PME/PDE (RT 0.434 vs. FB 0.489, *p* = 0.046). Bland–Altman analysis showed near‐zero fixed biases and no proportional bias, with limits of agreement from −0.53 to 0.62 (PME), −0.30 to 0.30 (Pi), −0.41 to 1.07 (PDE), −0.45 to 0.43 (α‐ATP), and −0.80 to 0.90 (NADPH).

**Data Conclusion:**

^31^P‐MRS of the liver showed equivalent stability and robustness for RT and FB. FB yielded comparable data within a shorter, predictable scan duration.

**Evidence Level:**

2.

**Technical Efficacy:**

1.

## Plain Language Summary

The liver plays a central role in the body's metabolism, and phosphorus magnetic resonance spectroscopy (^31^P‐MRS) is a noninvasive technique that can measure liver energy and membrane metabolism without a biopsy. A recently approved commercial coil now makes it possible to perform ^31^P‐MRS on standard clinical MRI systems at any facility. However, the optimal acquisition protocol for obtaining stable, high‐quality data within a clinically acceptable scan time has not yet been established. This study compared two approaches in 24 healthy volunteers, both designed to complete within approximately 13 min: respiratory triggering and free breathing. Both methods produced equivalent measurements of key liver metabolites, but free breathing consistently delivered a fixed, predictable scan duration, making it more practical for routine clinical use.

## 1 Introduction

Phosphorus magnetic resonance spectroscopy (^31^P‐MRS) allows noninvasive assessment of human tissue metabolism [1, 2, 3]. It permits the assessment of various phosphorus‐containing metabolites and their ratios, intracellular pH, and the kinetics of chemical reactions in energy metabolism [4, 5]. The liver is among the metabolic organs in the body, and it has been the topic of research using ^31^P‐MRS due to its large size and proximity to the body surface [4, 5]. Previous studies have reported that these metabolites are useful in diagnosing various diffuse liver disorders, including viral hepatitis [6], alcoholic liver disease [7, 8], metabolic dysfunction‐associated steatotic liver disease [9, 10], cirrhosis [11, 12, 13], and diabetes [14]. ^31^P‐MRS has been established as a powerful tool for noninvasive investigation of human liver metabolism under various physiological and pathophysiological conditions [5, 6, 7, 8, 9, 10, 11, 12, 13, 14].

Relative to proton (^1^H) spectroscopy, ^31^P‐MRS requires relatively large voxel sizes and longer measurement durations because of the lower sensitivity of ^31^P metabolites in vivo. Therefore, previous research on ^31^P‐MRS has been limited to facilities with access to dedicated coils and/or ultrahigh‐field MRI systems [4]. This limitation has hindered its application in general clinical practice.

Coils capable of acquiring ^31^P‐MRS data have recently received regulatory approval, and they are anticipated to accelerate the clinical application of ^31^P‐MRS. However, because the approved coil is a surface coil, optimization of the acquisition protocol is required to minimize signal contamination from the abdominal wall muscles and to ensure accurate, stable signal from the liver parenchyma [5, 15]. Hence, various localization techniques have been proposed for ^31^P‐MRS of the liver, including 2D/3D‐chemical shift imaging [16] for metabolic mapping [15, 17, 18], slab‐selective 1D image‐selected in vivo spectroscopy (ISIS) to reduce contamination [5], and single‐voxel 3D‐ISIS [19, 20] for fast and accurate localization. Each method has its advantages and disadvantages.

In general, the T2 relaxation times of ^31^P metabolites are relatively short [19]. To minimize the influence of T2 relaxation and J modulation on the acquired signals, “pulse‐acquire” or “non‐echo” free induction decay (FID)‐based MRS techniques are preferred [19]. Another important parameter to consider is the relatively large spectral dispersion of the ^31^P‐MR spectra. The only FID‐based single‐voxel spectroscopy (SVS) technique is ISIS. The chemical shift displacement error can lead to substantial bias in the localization accuracy of ^31^P spectra. Therefore, the selective inversion pulses are implemented with relatively large bandwidths to reduce the chemical shift displacement error [21]. Taking this into account and based on previous reports, high‐quality spectra with accurate spatial selectivity for the 3D volume can be acquired using the ISIS sequence within clinically acceptable measurement durations [21]. In addition, SVS localization sequences that allow full signal localization in one acquisition are preferred for liver parenchyma [5].

Given this, we believe that it is necessary to explore scanning methods that can efficiently and accurately acquire data from the liver parenchyma using coils that have received regulatory approval while ensuring reproducibility and robustness. There are two options for acquiring signals for single voxel ^31^P‐MRS using ISIS: respiratory‐triggering (RT) and free‐breathing (FB) techniques. The RT technique may lead to robust measurement; however, scan duration may be prolonged depending on the breathing pattern of the patient, which hampers the clinical workflow. The FB technique allows the acquisition of a greater number of signal averages within the same scan duration, but there is concern that noise and signal from outside the voxel of interest may be easily mixed in. The purpose of this study was to compare the data acquired using FB and RT with single‐voxel ^31^P‐MRS, each designed to achieve a similar expected scan duration of approximately 13 min for healthy volunteers.

## 2 Methods

This prospective study was approved by the Research Ethics Committee of our institution (Approval No. 24–032). Written informed consent was obtained from all participants prior to MRI.

### 2.1 Participants

Twenty‐four healthy participants aged 20–50 years (19 males and 5 females, mean age of 23.2 ± 0.8 years old) with no known history of abdominal disease were included in the study. All participants were confirmed to have no clinically relevant abnormalities based on health checkup results (including normal complete blood count, liver enzymes, serum lipids, and glycemic parameters). Their body composition measurements (height, body weight, body mass index, and body surface area) were within the normal ranges for the Japanese general population [22].

### 2.2 MR Data Acquisition

MRI was performed on a 3 T MR scanner with multinuclear capability (MR7700; Philips Healthcare, Best, the Netherlands) using the built‐in system body coil as transmit/receive for ^1^H imaging and for decoupling and nuclear Overhauser enhancement during spectroscopy, and a transmit/receive single loop ^31^P flex coil for ^31^P spectroscopy (Philips Healthcare, Best, the Netherlands). The ^31^P coil has received full regulatory approval, is available as product and can thus be used for routine clinical examinations. The ^31^P coil (Flex coil P‐140) is a flexible single‐loop transmit/receive surface coil with a square geometry of 14 × 14 cm, tuned to the ^31^P resonance frequency (51.7 MHz at 3 T). As a surface coil, the B1 field decreases with depth; adiabatic pulses were used to mitigate B1 inhomogeneity, and B1 calibration was performed using a spherical ^31^P phantom prior to each acquisition. The voxel of interest was positioned at a depth of up to approximately 7 cm from the coil surface. The scans were performed at 6:00 p.m. and the participants fasted for at least 5 h after having lunch between 12:00 p.m. and 1:00 p.m. ^1^H MR images for localization were obtained using the body coil. After obtaining scout images in three orientations (axial, coronal, and sagittal), T2‐weighted images were obtained with single‐shot spin‐echo images (repetition time [TR]/echo time [TE] = 8000/120 ms; field of view: 450 × 345 mm^2^) to determine the position of the diaphragm during scanning, to allow accurate identification of the liver position.

**FIGURE 1:**
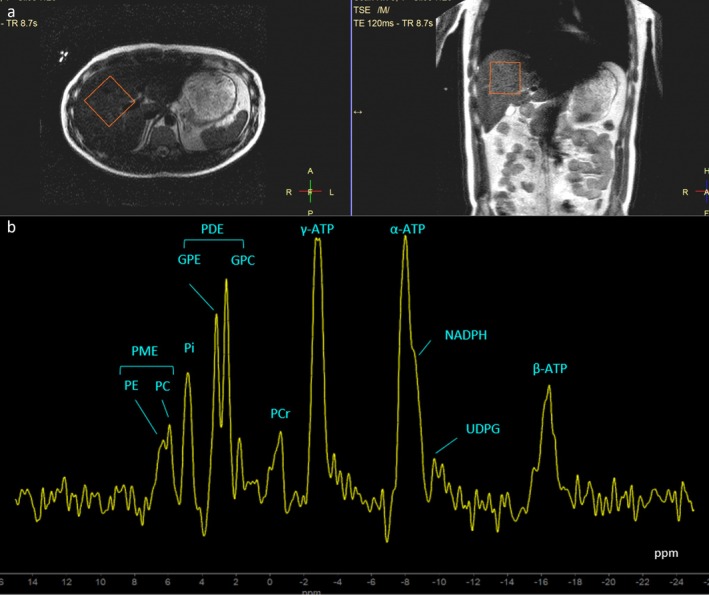
Axial and coronal images of the scan and the typical spectrum a. Placement of the voxel of interest in the axial and coronal T2‐weighted images. b. A typical liver phosphorus magnetic resonance spectrum of a healthy participant. Abbreviations: ATP = adenosine triphosphate, GPC = glycero‐phosphocholine, GPE = glycero‐phosphoethanolamine, NADPH = nicotinamide adenine dinucleotide phosphate, PC = phosphocholine, PCr = Phosphocreatine, PDE = phosphodiester, PE = phosphoethanolamine, Pi = inorganic phosphate, PME = phosphomonoester, UDPG = uridine diphosphoglucose.

A voxel of interest (55 × 55 × 55 mm^3^) was placed by two trained radiological technologists (with 42 and 13 years of clinical experience, respectively; both received dedicated multinuclear MRI training; acknowledged but not listed as co‐authors) within the liver to prevent immediate proximity to the lungs, gallbladder, abdominal muscle, primary branches of the portal vein, and the three major hepatic veins before ^31^P‐MRS acquisition (Figure 1).

A typical water line width of ∼28 Hz (range: 20–45 Hz) was obtained using automatic shimming with breath hold in exhaled state [23]. Three‐dimensionally localized liver spectra were obtained using ISIS [20] with a 5.42 ms hyperbolic secant adiabatic half‐passage pulse for excitation and a 4.22 ms hyperbolic secant full‐passage inversion pulse for slice selection (the hyperbolic secant inversion bandwidth = 2.95 kHz). The (minimum) TR was 4 s, and 2048 data points were acquired with a spectral resolution of 1.47 Hz/point. Proton decoupling was applied with broadband WALTZ‐4 pulse with an offset frequency of −0.8 Hz. Nuclear Overhauser enhancement was achieved via broadband saturation with a mixing time of 2700 ms and an offset frequency of −0.8 Hz. The transmitter frequency was centered on the γ‐ATP resonance (−2.5 ppm relative to PCr).

The number of signal averages, 128 for RT and 192 for FB, was chosen to achieve a clinically acceptable scan duration of approximately 13 min each, based on iterative pilot scanning that confirmed adequate spectral stability within this timeframe (Table 1). Because FB acquires data continuously without respiratory gating delays, more averages can be collected within the same nominal duration compared with RT. The actual signal acquisition times for both RT and FB were recorded and analyzed.

**TABLE 1:** Acquisition parameters.

Parameters	Respiratory triggering	Free breathing
Acquisition method	Single‐voxel ISIS	
Voxel size (FH/AP/RL)	55/55/55 mm^3^	
Repetition time (ms)	4000	
Proton decoupling	Broadband Waltz‐4	
NOE prepulse mix time (ms)	2700	
Respiratory comp.	Respiratory trigger	Free breathing
NSA	128	192
Spectral bandwidth (Hz)	3000	
Number samples	2048	
Expected examination time (min)	13	

Abbreviations: ISIS, image‐selected in vivo spectroscopy; NOE, nuclear Overhauser effect; NSA, number of signals averaged.

### 2.3 MR Spectroscopy Data Processing

All spectra were processed using the Java‐based MR user interface data analysis package (jMRUI version 7.0; http://www.mrui.uab.es/mrui/) [24]. They were apodized with a Gaussian line shape of 20 Hz and were manually zero order‐phased and frequency adjusted by a clinical scientist specializing in multinuclear MRI (J.K.) [9, 25, 26]. Peak fitting was performed using the advanced method for accurate, robust, and efficient spectral fitting (AMARES) [27, 28] algorithm in jMRUI. The algorithm uses a time‐domain fitting approach that incorporates prior knowledge to improve spectral fitting. It is most commonly used for the analysis of ^31^P‐MRS spectra because of the high flexibility of its fitting parameters. The assigned peaks were as follows: phosphomonoester (PME) resonance, inorganic phosphate (Pi), phosphodiester (PDE) resonance, which is split to glycero‐phosphoethanolamine (GPE) and glycero‐phosphocholine (GPC), phosphoenolpyruvate (PEP), phosphocreatine (PCr), γ‐adenosine triphosphate (ATP), α‐ATP, nicotinamide adenine dinucleotide phosphate (NADPH), uridine diphosphoglucose (UDPG), and β‐ATP peaks. PCr, UDPG, and β‐ATP peaks were not analyzed because these metabolites demonstrated very small peak areas in the liver due to limited abundance or reduced excitation by the adiabatic pulse [26, 29].

The signal‐to‐noise ratio (SNR) was used as an indicator of data quality and was calculated using the following formula [30]:

$$\mathrm{SNR}=\frac{\text{Magnitude of the maximum signal estimate in}\;\mathrm{FD}}{\text{noise}\;\mathrm{SD}\;\text{in}\;\mathrm{FD}}$$

where FD and SD represent Frequency Domain and standard deviation, respectively. In practice, the γ‐ATP peak had the maximum signal amplitude and was used for the SNR calculation.

### 2.4 Statistical Analysis

The PME–PDE (PME/PDE) and NADPH–PME and PDE ratios (NADPH/(PME + PDE)) were used to evaluate phosphorus metabolism in the liver [31]. Each metabolite peak area was normalized using the peak area of γ‐ATP (PME/γ‐ATP, Pi/γ‐ATP, PDE/γ‐ATP, α‐ATP/γ‐ATP, and NADPH/γ‐ATP) [32]. Continuous variables are expressed as mean ± SD. Data normality was confirmed using the Shapiro–Wilk test. The data for FB and RT were compared using a paired *t*‐test. Bland–Altman analysis was used to assess the agreement between the RT and FB results for each metabolite peak area normalized using the peak area of γ‐ATP [33]. For all statistical analyses, two‐sided *p* values < 0.05 were considered to indicate statistical significance. All statistical analyses were conducted using the SPSS software (version 27.0; IBM Corp., Armonk, NY, USA).

## 3 Results

Data from three participants were classified as low quality due to SNRs of 1.2 or below for either RT or FB and were excluded from the analysis because they were deemed not processable. One participant had only low‐quality RT data; one participant had only low‐quality FB data; and one participant had low‐quality RT and FB data. The number of nonanalyzable datasets was therefore 2 for RT and 2 for FB, confirming a symmetric failure rate between the two acquisition methods. Twenty‐one participants (15 males and 6 females) were included for the final analysis.

### 3.1 Participant Characteristics

The clinical and biochemical characteristics of the study participants are shown in Table 2. The physique data, complete blood count, and biochemistry results of all participants were within the normal ranges [22].

**TABLE 2:** Clinical data of the subjects.

Number of the subjects	21
Age	30.0 ± 6.9
Sex; male/female	15/6
Body composition	
Height (cm)	168.1 ± 7.5
Body weight (kg)	57.2 ± 9.2
Body mass index (kg/m^2^)	20.2 ± 2.7
Body surface area (m^2^)	1.64 ± 0.15
Liver enzymes	
Alanine aminotransferase (U/L)	16.5 ± 7.1
Aspartate aminotransferase (U/L)	19.9 ± 4.5
γ‐glutamyltransferase (U/L)	24.0 ± 13.8
Serum lipids	
Triglycerides (mmol/L)	73.5 ± 31.5
High‐density lipoprotein cholesterol (mmol/L)	66.7 ± 21.1
Low‐density lipoprotein cholesterol (mmol/L)	111.0 ± 26.9
Glycemic parameters	
Plasma glucose (mmol/L)	83.2 ± 6.1
Glycosylated hemoglobin (%)	5.2 ± 0.2
Complete blood count	
Hematocrit (%)	44.7 ± 3.2
Hematocrit (g/dL)	14.7 ± 1.2

*Note:* The blood test data for all participants are within the normal range.

### 3.2 Actual Scan Duration

The data acquisition duration was set at 12 min and 56 s during free breathing. The average actual signal acquisition duration using respiratory gating was 15 min and 44 s (range: 13 min 44–23 min 32 s). The actual scan duration was significantly longer for RT than for FB.

### 3.3 MR Spectroscopy Data and SNR

**FIGURE 2:**
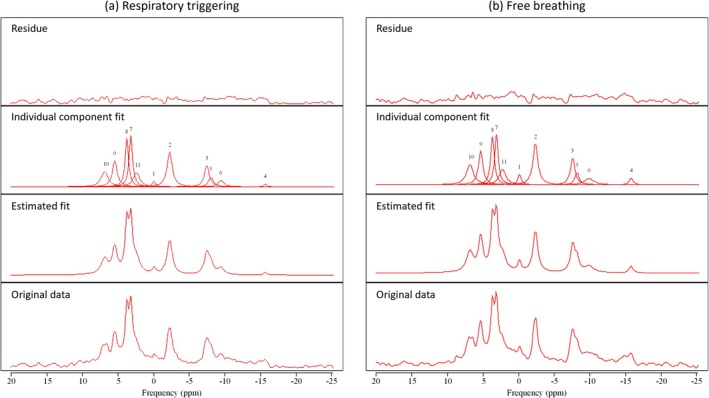
Representative spectra and spectral fitting results Representative spectra analyzed by jMRUI acquired with respiratory triggering (a) and free breathing (b). From bottom to top, the plots show the original data, the fitted spectrum (sum of all fitted components), the individual fitted peaks, and residual after the fitting. Each number represents the peak of a metabolite; 1 = Phosphocreatine (PCr), 2 = γ‐adenosine triphosphate (ATP), 3 = α‐ATP, 4 = β‐ATP, 5 = nicotinamide adenine dinucleotide phosphate (NADPH), 6 = uridine diphosphoglucose (UDPG), 7 = glycerophosphocholine (GPC), 8 = glycerophosphoethanolamine (GPE), 9 = inorganic phosphate (Pi), 10 = phosphomonoester (PME), 11 = phosphoenolpyruvate (PEP). The SNR for the spectrum with respiratory triggering (a) was 5.94, whereas that for free breathing (b) was 5.80.

**FIGURE 3:**
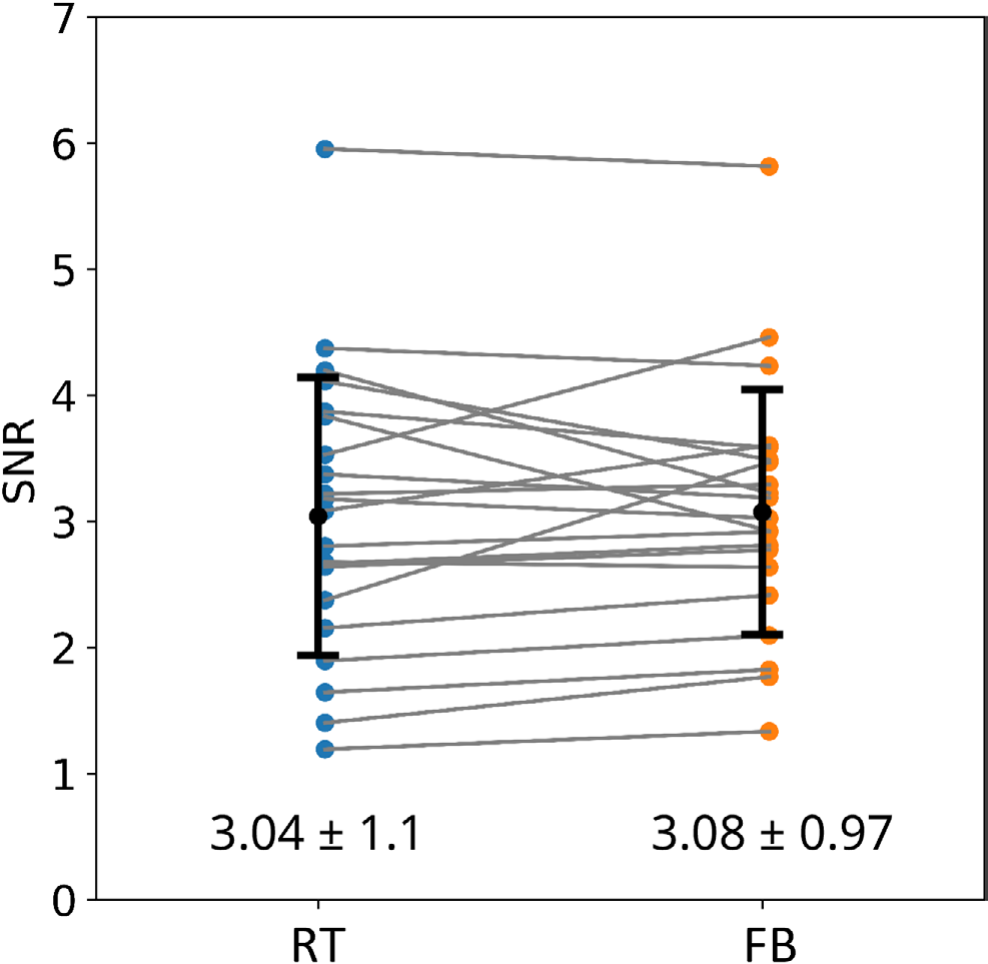
Comparison of the signal‐to‐noise ratio (SNR) No significant difference was found in the SNR between the scan methods with respiratory triggering (RT) and free breathing (FB).

No significant differences were observed between the RT and FB methods for NADPH/(PME + PDE) (*p* = 0.931), PME/γ‐ATP (*p* = 0.556), Pi/γ‐ATP (*p* = 0.931), α‐ATP/γ‐ATP (*p* = 0.332), and NADPH/γ‐ATP (*p* = 0.394) (Table 3). However, significant differences were noted between the RT and FB methods for PME/PDE and PDE/γ‐ATP (PME/PDE: RT 0.434 ± 0.314 versus FB 0.489 ± 0.291, *p* = 0.046; PDE/γ‐ATP: RT 1.68 ± 0.47 versus FB 1.35 ± 0.41, *p* = 0.003). Additionally, no significant difference was noted between the RT and FB methods for SNR (*p* = 0.570) (Figures 2 and 3; Table 3).

**TABLE 3:** MR spectroscopy data.

	Respiratory triggering	Free breathing	*p*
Ratio			
PME/PDE	0.434 ± 0.314	0.489 ± 0.291	0.046
NADPH/(PME + PDE)	0.312 ± 0.216	0.391 ± 0.396	0.931
PME/γ‐ATP	0.76 ± 0.64	0.72 ± 0.52	0.556
Pi/γ‐ATP	0.74 ± 0.34	0.74 ± 0.31	0.931
PDE/γ‐ATP	1.68 ± 0.47	1.35 ± 0.41	0.003
α‐ATP/γ‐ATP	0.66 ± 0.39	0.67 ± 0.35	0.332
NADPH/γ‐ATP	0.64 ± 0.29	0.59 ± 0.33	0.394
SNR	2.941 ± 1.151	2.989 ± 1.052	0.570

*Note:* No significant differences were observed between the respiratory triggering and free breathing methods for NADPH/(PME + PDE), PME/γ‐ATP, Pi/γ‐ATP, α‐ATP/γ‐ATP, and NADPH/γ‐ATP. Whereas, there were significant differences in PME/PDE and PDE/γ‐ATP between the respiratory triggering and free breathing methods. There was no significant difference in the SNR for the respiratory triggering and free breathing. Abbreviations: ATP = adenosine triphosphate, NADPH = nicotinamide adenine dinucleotide phosphate, PDE = phosphodiester, Pi = inorganic phosphate, PME = phosphomonoester, SNR = signal‐to‐noise ratio, UDPG = uridine diphosphoglucose.

**FIGURE 4:**
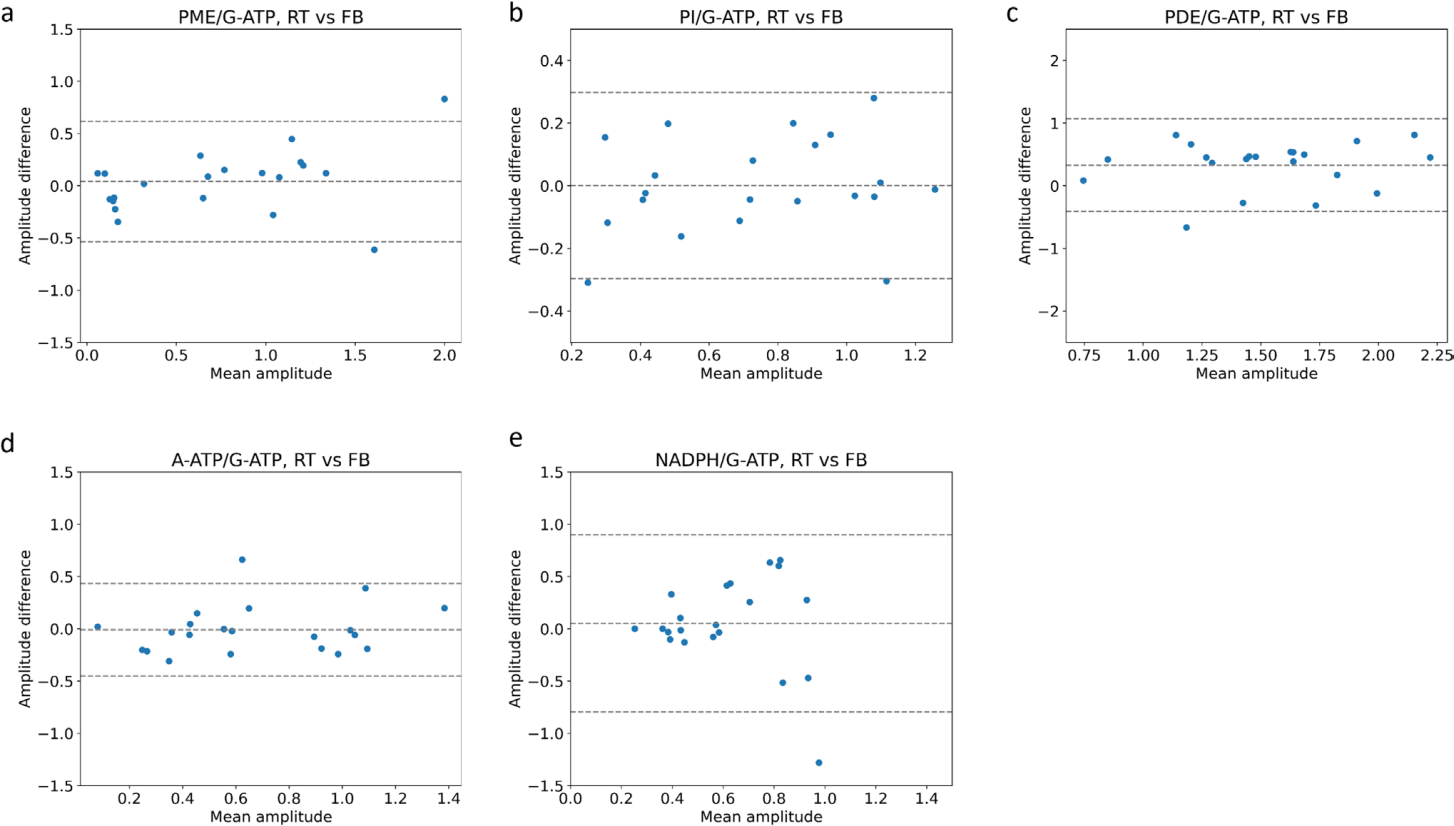
Bland–Altman plots of comparison of normalized metabolite peak area and Bland–Altman plots of metabolite amplitudes of phosphomonoester (PME: A), inorganic phosphate (Pi: B), phosphodiester (PDE: C), α‐adenosine triphosphate (ATP: D), and nicotinamide adenine dinucleotide phosphate (NADPH: E) normalized by the amplitude of γ‐ATP were compared between respiratory triggering (RT) and free breathing (FB). *Dashed lines = 95% limits of agreement.

The Bland–Altman analysis demonstrated moderate agreement between RT and FB acquisitions. The fixed biases observed were 0.04 for PME, 0.00 for Pi, 0.33 for PDE, −0.01 for α‐ATP, and 0.05 for NADPH. No proportional biases were seen (*p* = 0.084 for PME, 0.462 for Pi, 0.571 for PDE, 0.444 for α‐ATP, and 0.536 for NADPH). The limits of agreement as determined by twice the standard deviation were −0.53 to 0.62 for PME, −0.30 to 0.30 for Pi, −0.41 to 1.07 for PDE, −0.45 to 0.43 for α‐ATP, and −0.80 to 0.90 for NADPH (Figure 4).

## 4 Discussion

Our study revealed that the data from ^31^P‐MRS of the liver were not statistically significantly different for both RT and FB techniques. FB yielded comparable data within a shorter duration than RT. Additionally, the ability to obtain similar data using the two methods demonstrates the stability and robustness of data acquisition using a surface coil that has received regulatory approval.

Various previous studies have been conducted on ^31^P‐MRS of the liver [4, 5], but variations in the equipment or coils affect the obtained spectra. It is evident from the results of long‐term prospective studies using a modified coil [26, 34] that changes in the coil make it difficult to maintain consistency in the measurement of metabolites. Thus, verification using different coils with the same equipment has also been reported [26, 35]. Despite numerous reports on ^31^P‐MRS and its clinical value and utility [1, 2, 3, 4, 5, 6, 7, 8, 9, 10, 11, 12, 13, 14, 36], its generalizability remains low. However, the results obtained using regulatory‐approved equipment and coils hold considerable clinical relevance, as they can be directly applied across various facilities without the need for specialized hardware.

The coil used in this study has a circular ^31^P transmit‐receive single loop with a diameter of 14 cm. As mentioned previously, the consistency of the results requires careful validation when a new coil is introduced. Moreover, this regulatory‐approved coil is not specifically designed for the liver, and it is primarily a surface coil. Therefore, it is necessary to verify the most effective scanning for the liver. For diffuse liver disease assessment, where signals are acquired from the liver parenchyma rather than focal lesions, single‐voxel localization techniques are the standard approach. The common single‐voxel localization techniques are ISIS [20, 37], stimulated echo acquisition mode [37], and point‐resolved spectroscopy [37]. ISIS uses three frequency‐selective inversion pulses in the presence of three orthogonal magnetic field gradients. By switching the inversion pulses on and off, eight scans were obtained according to the encoding scheme. Adding and subtracting the different scans constructively adds signal from the desired location while canceling the signal from other locations. A disadvantage of ISIS is its sensitivity to motion, as eight scans must be obtained for full 3D localization [20]. ISIS is rarely used for ^1^H MRS because of potential artifacts such as those arising from incomplete water signal suppression and motion. Stimulated echo acquisition mode and point‐resolved spectroscopy are the most common single‐voxel localization techniques for ^1^H MRS. However, ISIS is the preferred method for ^31^P‐MRS localization as effects due to the relatively rapid T2 decay and J‐coupling are avoided [21]. ISIS has been employed for ^31^P‐MRS signal acquisition from the liver parenchyma in patients with hepatitis [6], metabolic dysfunction‐associated steatotic liver disease [9, 10], and type 2 diabetes [14], and we have also utilized this method.

In this study, we adjusted the parameters to align with a similar expected examination time, considering the workflow in a clinical setting. Despite this adjustment, an extension of the signal acquisition duration was observed in some volunteers when using the RT method, resulting in a notably longer examination duration. There are individual differences in breathing intervals, and it takes more time for individuals with longer breathing intervals to acquire data. Second, data acquisition may be skipped during respiratory synchronization depending on the breathing pattern. Consequently, the actual scan duration is prolonged beyond the predefined scan time, making it difficult to anticipate the completion time. This complicates the workflow and poses challenges for use in clinical settings. In contrast, data are continuously acquired with the FB method, eliminating the need to extend imaging time and ensuring a fixed examination duration. This makes it more feasible in routine clinical practice if equivalent data can be obtained.

Most ^31^P‐MRS metrics of the liver parenchyma were not statistically significantly different between RT and FB techniques. While the limits of agreement in Bland–Altman analysis represent approximately 50% of the mean amplitude, this level of variability is considered acceptable given several considerations. First, the near‐zero fixed biases and absence of proportional bias indicate that FB does not systematically over‐ or underestimate metabolite concentrations compared to RT. Second, this degree of variation is expected given the inherently low SNR of the data. Notably, no significant difference was observed for NADPH/(PME + PDE), suggesting that the NADPH/(PME + PDE) ratio is unlikely to pose issues for clinical application, regardless of which acquisition method is used.

However, significant differences were found in PME/PDE and PDE/γ‐ATP. It has been reported that PME and PDE peak areas can be difficult to quantify because of overlap with other peaks and baseline fluctuations [38, 39]. The ratio of peak areas was chosen because it normalizes variations in measurement conditions, enabling comparisons across time points and participants. The PDE/γ‐ATP values obtained with RT were notably larger than those with FB. Although γ‐ATP normalization is often preferred because the γ‐ATP peak is well‐defined and less prone to overlap [32], ATP levels can be affected by fasting duration, exercise, and various pathologies, and the γ‐ATP peak may be saturated in some pulse sequences. PME/γ‐ATP may be valuable for studies evaluating the relationship between energy metabolism and membrane synthesis [8, 31], whereas PME/PDE is more suited for assessment of membrane turnover and is especially relevant for cancer research and liver disease studies [30, 31]. Both indicators have also been applied in the assessment of viral hepatitis [6], alcoholic liver disease [8], metabolic dysfunction‐associated steatotic liver disease [9], and liver cirrhosis [12], but robust data were not obtained in the present healthy volunteer cohort. Further studies involving larger and more diverse samples are warranted.

Among the three cases with low SNR and data that were not processable (SNR < 1.2), data from two cases using RT and one case using FB could not be analyzed. It is presumed that data quality is influenced by both breathing conditions and body composition. This study on healthy volunteers did not include individuals who were unable to maintain consistent breathing patterns. However, measurements of body composition in people with obesity using a single‐loop coil are often challenging, as there can be a large distance between the coil and the voxel of interest. This distance results in a reduced SNR and potentially poor‐quality spectra. In individuals with a very lean body type and small waist circumference, the rigid material of the coil may not conform well to the abdomen of the participant, potentially resulting in a large gap between the coil and the body. Although this study did not happen to include individuals with extreme obesity or an extremely lean body habitus, such body compositions may contribute to poor data quality in clinical settings, and the reasons for the poor data in these three cases remain unclear.

### 4.1 Limitations

First, our study involved only healthy volunteers with adequate breath‐holding ability and stable breathing. Therefore, the clinical usefulness of the proposed method requires further investigation given that patients of different ages with various abdominal diseases are examined in clinical practice. Patients with certain conditions may breathe less regularly than healthy volunteers, and motion‐related signal corruption may therefore occur more frequently in clinical settings. Consequently, implementing a data quality management strategy may be necessary for routine clinical use. For example, signal quality could be monitored for each acquisition block (or frame), and segments with insufficient SNR or evident motion contamination could be excluded prior to re‐averaging and refitting. Second, this study focused on one acquisition technique (ISIS) with fixed parameters, apart from the number of signal averages and respiratory acquisition strategy. Evaluating additional parameters, such as voxel size, repetition time, echo time, and the presence or absence of proton decoupling, can enhance the robustness and repeatability of the data. Chemical shift imaging techniques, rather than ISIS, are often used to target liver tumors. Therefore, further studies are warranted. Third, despite being healthy volunteers with stable breathing, there was considerable variability in SNR and other values, and the cause of this variability remains unclear. Further validation of the data stability is necessary. Fourth, the number of signal averages was determined empirically based on pilot observations rather than formal power analysis; systematic optimization of acquisition parameters warrants future investigation. Fifth, absolute metabolite concentrations were not validated against an external reference standard such as phantom‐based quantification, which was beyond the scope of the present study. Sixth, scan‐rescan reproducibility was not assessed. As this is, to our knowledge, the first study using this regulatory‐approved coil for liver ^31^P‐MRS, a within‐session comparison between RT and FB represents a necessary first step; formal test–retest assessment remains an important future direction.

Despite these limitations, the FB method is favored for routine clinical use for the following reasons [1]: it achieves statistically equivalent results for the clinically important metrics NADPH/(PME + PDE), PME/γ‐ATP, Pi/γ‐ATP, α‐ATP/γ‐ATP, and NADPH/γ‐ATP [2]; it provides a fixed, predictable acquisition time of approximately 13 min, in contrast to RT, whose actual duration varied substantially (range: 13 min 44 s–23 min 32 s); and [3] the rate of nonanalyzable data was equal between the two methods. Although a significant difference was observed for PDE/γ‐ATP, PDE quantification is inherently challenging due to peak overlap and baseline fluctuations, and the clinically relevant NADPH/(PME + PDE) ratio showed no significant difference. Overall, the evidence supports FB as the preferred acquisition approach for clinical liver ^31^P‐MRS protocols.

## 5 Conclusion

This study prospectively compared respiratory‐triggered (RT) and free‐breathing (FB) signal acquisition methods for single‐voxel ^31^P‐MRS of the liver using a regulatory‐approved surface coil on a clinical 3 T MRI system. The majority of ^31^P‐MRS metrics, including the clinically relevant NADPH/(PME + PDE) ratio, were not statistically significantly different between the two acquisition methods. FB yielded comparable data within a shorter and predictable scan duration of approximately 13 min, compared with RT, whose actual scan duration was variable and notably longer. The near‐zero fixed biases and absence of proportional bias in Bland–Altman analysis further support the equivalence of the two methods for most metabolite measurements. These findings suggest that FB acquisition is a practical and clinically feasible approach for liver ^31^P‐MRS using regulatory‐approved coils, potentially facilitating its broader implementation in routine clinical practice.

## Funding

The authors have no relevant financial or nonfinancial interests to disclose.

## Ethics Statement

The institutional review board of our institution approved this prospective study (Approval No. 24–032), and the written informed consent to participate in the study was obtained from all participants. The study was conducted in accord with the ethical standards of the IRB and with the 1964 Helsinki Declaration and its later amendments.

## Data Availability

The datasets used and/or analyzed during the current study are available from the corresponding author on reasonable request.

## References

[1] J. J. Ackerman , T. H. Grove , G. G. Wong , D. G. Gadian , and G. K. Radda , “Mapping of Metabolites in Whole Animals by 31P NMR Using Surface Coils,” Nature 283 (1980): 167–170. 7350541 10.1038/283167a0

[2] B. Chance , S. Eleff , J. S. Leigh, Jr. , D. Sokolow , and A. Sapega , “Mitochondrial Regulation of Phosphocreatine/Inorganic Phosphate Ratios in Exercising Human Muscle: A Gated 31P NMR Study,” Proceedings of the National Academy of Sciences of the United States of America 78 (1981): 6714–6718. 6947247 10.1073/pnas.78.11.6714 PMC349120

[3] J. J. Prompers , J. A. Jeneson , M. R. Drost , C. C. Oomens , G. J. Strijkers , and K. Nicolay , “Dynamic MRS and MRI of Skeletal Muscle Function and Biomechanics,” NMR in Biomedicine 19 (2006): 927–953. 17075956 10.1002/nbm.1095

[4] L. Valkovic , M. Chmelik , and M. Krssak , “In‐Vivo 31P‐MRS of Skeletal Muscle and Liver: A Way for Non‐Invasive Assessment of Their Metabolism,” Analytical Biochemistry 529 (2017): 193–215. 28119063 10.1016/j.ab.2017.01.018 PMC5478074

[5] A. I. Schmid , M. Chmelik , J. Szendroedi , et al., “Quantitative ATP Synthesis in Human Liver Measured by Localized P‐31 Spectroscopy Using the Magnetization Transfer Experiment,” NMR in Biomedicine 21 (2008): 437–443. 17910026 10.1002/nbm.1207

[6] A. K. Lim , N. Patel , G. Hamilton , et al., “31P MR Spectroscopy in Assessment of Response to Antiviral Therapy for Hepatitis C Virus‐Related Liver Disease,” American Journal of Roentgenology 189 (2007): 819–823. 17885051 10.2214/AJR.07.2418

[7] D. J. Meyerhoff , M. D. Boska , A. M. Thomas , and M. W. Weiner , “Alcoholic Liver Disease: Quantitative Image‐Guided P‐31 MR Spectroscopy,” Radiology 173 (1989): 393–400. 2798871 10.1148/radiology.173.2.2798871

[8] D. K. Menon , M. Harris , J. Sargentoni , S. D. Taylor‐Robinson , I. J. Cox , and M. Y. Morgan , “In Vivo Hepatic 31P Magnetic Resonance Spectroscopy in Chronic Alcohol Abusers,” Gastroenterology 108 (1995): 776–788. 7875480 10.1016/0016-5085(95)90451-4

[9] K. Sevastianova , A. Hakkarainen , A. Kotronen , et al., “Nonalcoholic Fatty Liver Disease: Detection of Elevated Nicotinamide Adenine Dinucleotide Phosphate With In Vivo 3.0‐T 31P MR Spectroscopy With Proton Decoupling,” Radiology 256 (2010): 466–473. 20656836 10.1148/radiol.10091351

[10] L. Valkovič , M. Gajdošík , S. Traussnigg , et al., “Application of Localized 31P MRS Saturation Transfer at 7 T for Measurement of ATP Metabolism in the Liver: Reproducibility and Initial Clinical Application in Patients With Non‐Alcoholic Fatty Liver Disease,” European Radiology 24 (2014): 1602–1609. 24647824 10.1007/s00330-014-3141-x

[11] D. K. Menon , J. Sargentoni , S. D. Taylor‐Robinson , et al., “Effect of Functional Grade and Etiology on In Vivo Hepatic Phosphorus‐31 Magnetic Resonance Spectroscopy in Cirrhosis: Biochemical Basis of Spectral Appearances,” Hepatology 21 (1995): 417–427. 7843715

[12] M. Dezortová , P. Taimr , A. Škoch , J. Spicák , and M. Hájek , “Etiology and Functional Status of Liver Cirrhosis by 31P MR Spectroscopy,” World Journal of Gastroenterology 11 (2005): 6926–6931. 16437594 10.3748/wjg.v11.i44.6926 PMC4717032

[13] S. D. Taylor‐Robinson , J. Sargentoni , J. D. Bell , et al., “In Vivo and In Vitro Hepatic 31P Magnetic Resonance Spectroscopy and Electron Microscopy of the Cirrhotic Liver,” Liver 17 (1997): 198–209. 9298490 10.1111/j.1600-0676.1997.tb00806.x

[14] A. I. Schmid , J. Szendroedi , M. Chmelik , M. Krssak , E. Moser , and M. Roden , “Liver ATP Synthesis Is Lower and Relates to Insulin Sensitivity in Patients With Type 2 Diabetes,” Diabetes Care 34 (2011): 448–453. 21216854 10.2337/dc10-1076 PMC3024365

[15] M. Chmelik , A. I. Schmid , S. Gruber , et al., “Three‐Dimensional High‐Resolution Magnetic Resonance Spectroscopic Imaging for Absolute Quantification of 31P Metabolites in Human Liver,” Magnetic Resonance in Medicine 60 (2008): 796–802. 18816829 10.1002/mrm.21762

[16] T. R. Brown , B. M. Kincaid , and K. Ugurbil , “NMR Chemical Shift Imaging in Three Dimensions,” Proceedings of the National Academy of Sciences of the United States of America 79 (1982): 3523–3526. 6954498 10.1073/pnas.79.11.3523 PMC346453

[17] A. Panda , S. Jones , H. Stark , et al., “Phosphorus Liver MRSI at 3 T Using a Novel Dual‐Tuned Eight‐Channel 31P/1H Coil,” Magnetic Resonance in Medicine 68 (2012): 1346–1356. 22287206 10.1002/mrm.24164

[18] I. J. Cox , D. J. Bryant , A. G. Collins , et al., “Four‐Dimensional Chemical Shift MR Imaging of Phosphorus Metabolites of Normal and Diseased Human Liver,” Journal of Computer Assisted Tomography 12 (1988): 369–376. 3366944 10.1097/00004728-198805010-00001

[19] W. Bogner , M. Chmelik , A. I. Schmid , E. Moser , S. Trattnig , and S. Gruber , “Assessment of 31P Relaxation Times in the Human Calf Muscle: A Comparison Between 3 T and 7 T In Vivo,” Magnetic Resonance in Medicine 62 (2009): 574–582. 19526487 10.1002/mrm.22057

[20] R. J. Ordidge , A. Connelly , and J. A. B. Lohman , “Image‐Selected In Vivo Spectroscopy (ISIS). A New Technique for Spatially Selective Nmr Spectroscopy,” Journal of Magnetic Resonance 66 (1986): 283–294.

[21] W. Bogner , M. Chmelik , O. C. Andronesi , A. G. Sorensen , S. Trattnig , and S. Gruber , “In Vivo 31P Spectroscopy by Fully Adiabatic Extended Image Selected In Vivo Spectroscopy: A Comparison Between 3 T and 7 T,” Magnetic Resonance in Medicine 66 (2011): 923–930. 21446033 10.1002/mrm.22897

[22] Ministry of Internal Affairs and Communications , Statistics Bureau. e‐Stat: Portal Site of Official Statistics of Japan. accessed August, 2025, https://www.e‐stat.go.jp.

[23] R. Gruetter , “Automatic Localized In Vivo Adjustment of All First‐ and Second‐Order Shim Coils,” Magnetic Resonance in Medicine 29 (1993): 804–811. 8350724 10.1002/mrm.1910290613

[24] A. Naressi , C. Couturier , J. M. Devos , et al., “Java‐Based Graphical User Interface for the MRUI Quantitation Package,” Magma 12 (2001): 141–152. 11390270 10.1007/BF02668096

[25] C. W. Li , W. G. Negendank , J. Murphy‐Boesch , K. Padavic‐Shaller , and T. R. Brown , “Molar Quantitation of Hepatic Metabolites In Vivo in Proton‐Decoupled, Nuclear Overhauser Effect Enhanced 31P NMR Spectra Localized by Three‐Dimensional Chemical Shift Imaging,” NMR in Biomedicine 9 (1996): 141–155. 9015801 10.1002/(SICI)1099-1492(199606)9:4<141::AID-NBM403>3.0.CO;2-P

[26] M. Jonuscheit , S. Wierichs , M. Rothe , et al., “Reproducibility of Absolute Quantification of Adenosine Triphosphate and Inorganic Phosphate in the Liver With Localized 31P‐Magnetic Resonance Spectroscopy at 3‐T Using Different Coils,” NMR in Biomedicine 37 (2024): e5120. 38404058 10.1002/nbm.5120

[27] S. Mierisová , A. van den Boogaart , I. Tkác , P. van Hecke , L. Vanhamme , and T. Liptaj , “New Approach for Quantitation of Short Echo Time In Vivo 1H MR Spectra of Brain Using AMARES,” NMR in Biomedicine 11 (1998): 32–39. 9608586 10.1002/(sici)1099-1492(199802)11:1<32::aid-nbm501>3.0.co;2-#

[28] L. Vanhamme , A. van den Boogaart , and S. van Huffel , “Improved Method for Accurate and Efficient Quantification of MRS Data With Use of Prior Knowledge,” Journal of Magnetic Resonance 129 (1997): 35–43. 9405214 10.1006/jmre.1997.1244

[29] A. Laufs , R. Livingstone , B. Nowotny , et al., “Quantitative Liver 31P Magnetic Resonance Spectroscopy at 3T on a Clinical Scanner,” Magnetic Resonance in Medicine 71 (2014): 1670–1675. 23798380 10.1002/mrm.24835

[30] R. Kreis , V. Boer , I.‐Y. Choi , et al., “Experts' Working Group on Terminology for MR Spectroscopy Terminology and Concepts for the Characterization of In Vivo MR Spectroscopy Methods and MR Spectra: Background and Experts' Consensus Recommendations,” NMR in Biomedicine 34 (2020): e4347. 32808407 10.1002/nbm.4347 PMC7887137

[31] L. W. F. Seelen , L. van den Wildenberg , W. J. M. van der Kemp , et al., “Prospective of 31P MR Spectroscopy in Hepatopancreatobiliary Cancer: A Systematic Review of the Literature,” Journal of Magnetic Resonance Imaging 57 (2023): 1144–1155. 35916278 10.1002/jmri.28372

[32] S. Traussnigg , C. Kienbacher , M. Gajdošík , et al., “Ultra‐High‐Field Magnetic Resonance Spectroscopy in Non‐Alcoholic Fatty Liver Disease: Novel Mechanistic and Diagnostic Insights of Energy Metabolism in Non‐Alcoholic Steatohepatitis and Advanced Fibrosis,” Liver International 37 (2017): 1544–1553. 28544208 10.1111/liv.13451 PMC5638103

[33] J. M. Bland and D. G. Altman , “Statistical Methods for Assessing Agreement Between Two Methods of Clinical Measurement,” Lancet 1 (1986): 307–310. 2868172

[34] J. Szendroedi , A. Saxena , K. S. Weber , et al., “Cohort Profile: The German Diabetes Study (GDS),” Cardiovascular Diabetology 15 (2016): 59. 27053136 10.1186/s12933-016-0374-9 PMC4823856

[35] M. Wylezinska , J. F. Cobbold , J. Fitzpatrick , et al., “A Comparison of Single‐Voxel Clinical In Vivo Hepatic 31P MR Spectra Acquired at 1.5 and 3.0 Tesla in Health and Diseased States,” NMR in Biomedicine 24 (2011): 231–237. 20949641 10.1002/nbm.1578

[36] M. Chmelik , M. Považan , M. Krššák , et al., “In Vivo (31)P Magnetic Resonance Spectroscopy of the Human Liver at 7 T: An Initial Experience,” NMR in Biomedicine 27 (2014): 478–485. 24615903 10.1002/nbm.3084

[37] E. G. ter Voert , L. Heijmen , H. W. van Laarhoven , and A. Heerschap , “In Vivo Magnetic Resonance Spectroscopy of Liver Tumors and Metastases,” World Journal of Gastroenterology 17 (2011): 5133–5149. 22215937 10.3748/wjg.v17.i47.5133 PMC3243879

[38] P. Sedivy , T. Dusilova , M. Hajek , M. Burian , M. Krššák , and M. Dezortova , “In Vitro 31P MR Chemical Shifts of In Vivo‐Detectable Metabolites at 3T as a Basis Set for a Pilot Evaluation of Skeletal Muscle and Liver 31P Spectra With LCModel Software,” Molecules 26 (2021): 7571. 34946652 10.3390/molecules26247571 PMC8703310

[39] L. A. B. Purvis , W. T. Clarke , L. Valkovič , et al., “Phosphodiester Content Measured in Human Liver by In Vivo 31 P MR Spectroscopy at 7 Tesla,” Magnetic Resonance in Medicine 78 (2017): 2095–2105. 28244131 10.1002/mrm.26635 PMC5697655

